# Does comsumption of staining drinks compromise the result of tooth whitening?

**DOI:** 10.4317/jced.56316

**Published:** 2019-11-01

**Authors:** Jhones-Suelone-Pontes Nogueira, Paulo-Cardoso Lins-Filho, Marlon-Ferreira Dias, Marianna-Falcão Silva, Renata-Pedrosa Guimarães

**Affiliations:** 1 Graduated in Dentistry from Federal University of Pernambuco; 2 Master student of the Dentistry postgraduate program of Federal University of Pernambuco; 3 Professor at Federal University of Pernambuco

## Abstract

**Background:**

After dental bleaching procedures dentists commonly advise patients to reduce the consumption of beverages that may cause the teeth to stain, however, the effectiveness of teeth whitening may not be directly affected by diet.

**Material and Methods:**

It was evaluated through in vitro study whether contact with dyes through in-office bleaching sessions with 35% hydrogen peroxide would influence the effectiveness of treatment. Sixty bovine incisors were randomly assigned into 5 groups (n = 12) according to contact frequency and type of dye solutions. All dental elements received three in-office bleaching sessions with 35% hydrogen peroxide one week apart. Except for GCTRL (control), all experimental groups were submerged in dyes (coffee or wine) for 5 min once a day. In groups GC24 and GW24 contact with the dyes was made from 24 hours after each bleaching session, while in groups GC72 and GW72, from 72 hours. The color was measured with a digital spectrophotometer. Data were expressed as statistics: mean and standard deviation.

**Results:**

Contact with dyes during in-office bleaching treatment with 35% hydrogen peroxide did not influence the staining averages after three bleaching sessions. The speed of the whitening effect was influenced by contact with coffee from 24 hours after the sessions and with wine from 24 hours and 72 hours after the whitening session. The whitening result was reversed after one week for all groups, especially for groups that came in contact with red wine either 24 hours or 72 hours after session and coffee after 24 hours.

**Conclusions:**

Contact with dyes during in-office bleaching treatment did not influence the final staining averages after three bleaching sessions although there was influence on speed of the whitening effect between the sessions.

** Key words:**Tooth bleaching, hydrogen peroxide, spectrophotometry.

## Introduction

Facial attraction, which includes a harmonic and aesthetic smile, is a crucial factor in social interaction (1). During social interaction, the speaker’s mouth and eyes are the main points the listener directs his attention to. In addition, tooth color and gingival display are fundamental factors for analyzing a good smile appearance (2).

The dental bleaching treatment has been much requested by patients, because white teeth are considered healthy and beautiful (3). The change in the color of dental elements is the result of physical and chemical interaction between dental tissues and may be caused by extrinsic or intrinsic factors. The main products and foods that cause extrinsic pigmentation include coffee, black tea, tobacco, red wines and cola drinks (3-5).

Tooth whitening can be performed with hydrogen peroxide or carbamide peroxide, which are effective at different concentrations. It can be supervised, using trays, in the office with or without light activation. For light to be used safely, a device with lower power density and spacing of activations should be used, so that there is cooling time of the dental structure and lower risk of postoperative sensitivity and pulp problems (5-9).

Hydrogen peroxide can be applied directly, or it can be produced from carbamide peroxide. It penetrates the tooth and produces free radicals, which then attack and break dark-colored molecules6. Dentists advise patients to reduce coffee and tea consumption, and to avoid smoking or any other habit that may cause tooth staining, especially after bleaching, as some studies have reported that bleaching agents may alter the surface texture and morphology of the tooth. enamel, making it more susceptible to dye absorption (3,4,10,11).

However, more recent studies state that the effectiveness of teeth whitening may not be directly affected by diet (3,4,12,13). Most of these studies were performed using the supervised whitening technique only and there is no consensus on these findings. In this sense, the objective of the present study was to evaluate, in vitro, if the contact with dyes will influence the result of in-office whitening.

## Material and Methods

This study was submitted and approved by the Animal Use Ethics Committee of the Health Sciences Center of the Federal University of Pernambuco, CEUA: 23076.042700 / 2016-69).

Sixty bovine incisors were selected, with coronary integrity (absence of wear facets or pathological alterations of enamel, observed in stereoscopic magnifying glass), which were submerged in a 0.5% chloramine solution for 7 days for disinfection. Extrinsic spots and organic deposits were removed by curette scraping, pumice paste prophylaxis and Robinson brush. After washing in running water, they were stored in 0.9% physiological solution, under refrigeration.

Dental elements were sectioned 1mm beyond the cementoenamel junction, pulp remains were excised and the root canal entrance sealed with composite resin. They were then randomly distributed into groups according to the frequency of contact and the type of dye solutions ( Table 1).

**Table 1:**
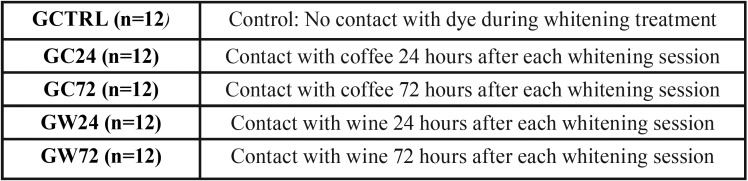
Characterization of experimental groups.

All elements received three in-office bleaching sessions with 35% hydrogen peroxide (Whiteness HP 35% / FGM) one week apart. Between sessions, teeth were placed in containers containing artificial saliva in a biological oven at 37ºC. In each session a single application of the gel was performed for 40 minutes following the manufacturer’s protocol:

1) Prophylaxis with pumice stone and Robinson brush

2) Initial color registration

3) Gel preparation: in the ratio of 3 drops of peroxide to 1 drop of dispersant

4) Apply a layer of gel over the entire buccal surface of the teeth to be whitened. The gel layer should be 0.5 to 1mm thick;

5) Gel rests for 40 minutes;

6) Gel’s Aspiration with a surgical cannula and washing the teeth with distilled water in abundance;

7) Surface polishing with Diamond Excel (FGM) polishing paste and Diamond felt disc (FGM);

8) Application of FlugeNeutral Topical Fluoride (DFL) for 4 minutes;

9) Color registration (Easy Shade / VITA Digital Spectrophotometer).

Except for the control group, all experimental groups were submerged in their dyes for 5 min once a day. Groups GC24 and GW24 contact with the dyes was made from 24 hours after each bleaching session, in groups GC72 and GW72, from 72 hours after each bleaching session.

Color assessment was performed at the following times:

1. TBase: Before Whitening

2. T1: After the first whitening session

3. T2: After prophylaxis and before the second whitening session

4. T3: After the second whitening session

5. T4: After prophylaxis and before the third whitening session

6. T5: After the third whitening session

7. T6: One week after the third whitening session before prophylaxis

8. T7: One week after the third whitening session after prophylaxis

Color was measured with a portable digital spectrophotometer (Easyshade-Vita, Brea, California, USA) according to the CIE Lab system3,8,9,14. The color of the middle third of the teeth was taken as reference. Calibration of the device has always been performed before each measurement. To standardize the color measurement site, a thermoplasticized 2mm Ethylene / Vinyl Acetate copolymer (Whiteness –FGM) matrix was made over the teeth.

The matrix was drilled in the middle third in the buccal region of the incisors with the aid of a 6 mm circular scalpel (similar to the active tip of the spectrophotometer).

To determine the color differences at different times of treatment, ΔE was calculated using the formula: ΔE * = [ΔL * 2 + Δa2 + Δb2] 1/2, where ΔL * = L0-L1; Δa = a0-a1; Δb = b0-b1.

**Figure 1:**
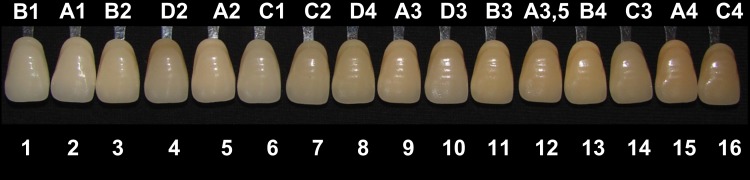
Numerical values corresponding to the Classic Vita Scale in order of brightness.

For analysis of the bleaching speed, the values provided by the spectrophotometer in the Vita Classical scale, converted into numerical values according to the degree of luminosity, as seen on Fig. 1.

Data were expressed as statistics: mean and standard deviation. For comparison between groups at each time, the F (ANOVA) or Kruskal-Wallis test and the paired T-test were used to compare Delta 6 (T5-TBASE) and Delta 9 (T7 - TBASE).

It is noteworthy that in case of significant difference Tukey’s multiple comparisons tests (between group pairs) were used when the F test (ANOVA) was used and Kruskal-Wallis comparisons when the test was used. The F (ANOVA) and paired Student t-tests were used in situations where data normality was verified and the Kruskal-Walis test in case of normality rejection. Data normality was verified by the Shapiro-Wilk test.

The margin of error used in deciding statistical tests was 5.0%. Data were entered into an EXCEL table and then transported for statistical analysis in SPSS (IBM-SPSS Statistics, version 23).

## Results

 Table 2 shows the statistics obtained with the average values of color variations (ΔE) at different times. To evaluate the color variation between the sessions and the whitening result, we considered ΔE1, ΔE3, ΔE5 and ΔE6 (marked in blue). It can be observed that in all analyzes, there was significant difference only in ΔE5 (color difference between the third and second bleaching session), with a higher mean for the GW72 group.

**Table 2:**
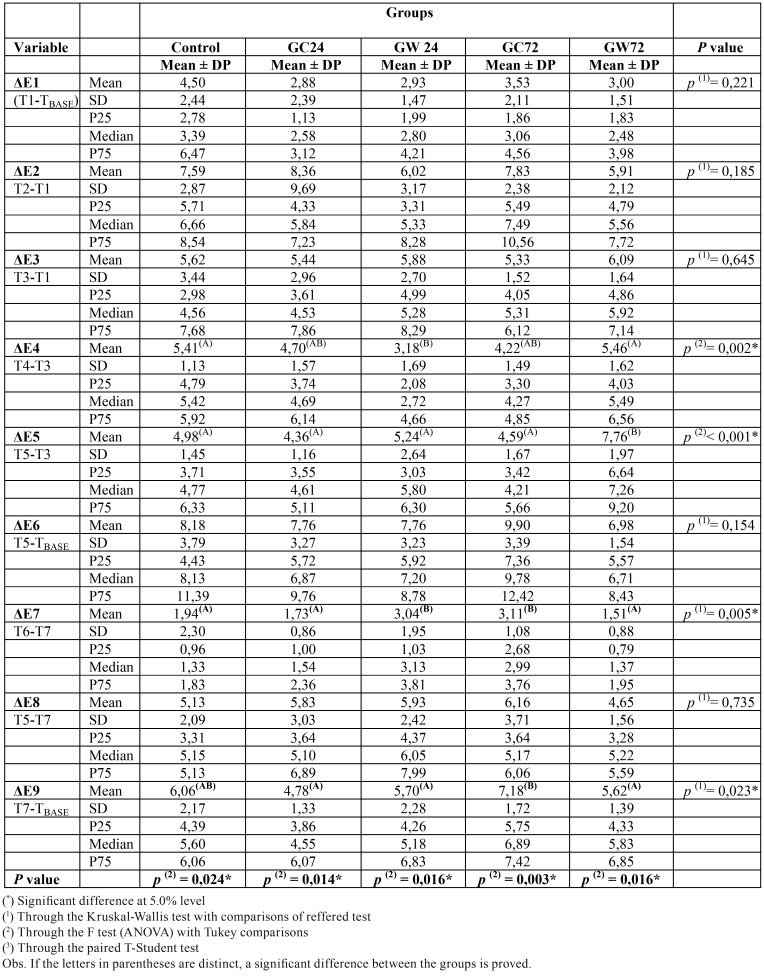
Mean ΔE values according to group.

By highlighting the ΔE2, ΔE4, ΔE8 (marked in green), the influence of daily contact with the dyes is observed between the whitening sessions. According to the results, there were no significant differences between groups, except for ΔE4 (color difference between the end of the second session and before the third session), where GW24 obtained the smallest color variation.

 Table 2 also reveals that when analyzing ΔE9, the overall result was not changed after one week, although a slight reversal of results was observed for all groups, then: it was possible to verify that the means were correspondingly higher in ΔE6 compared to ΔE9. In this evaluation time, there were significant differences with higher color variation value for group GC72, compared to the others.

**Figure 2:**
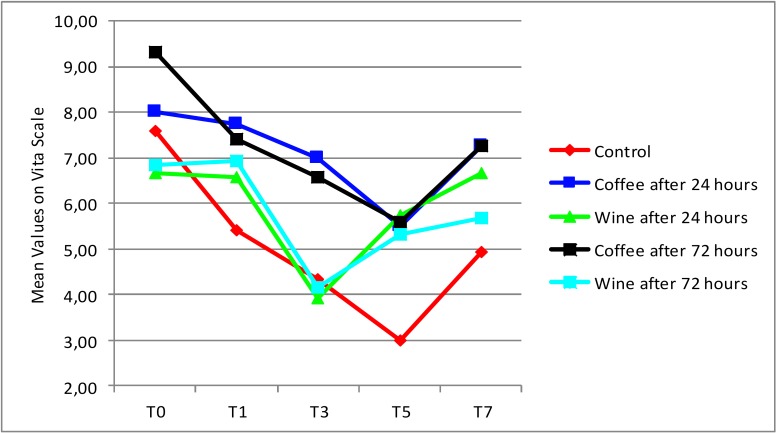
Lightening evolution according to the mean values based on the Classic Vita scale in order of brightness.

Fig. 2 shows the evolution of bleaching according to the mean values based on the Classic Vita scale in order of brightness. In all groups the means were correspondingly higher at baseline (T0) than in the other evaluations; except for the GW72 group whose mean had a slight increase from baseline to T1. This chart also shows the reversal of whitening results after one week for all groups.

## Discussion

Color is a perception matter and subjective in its interpretation. Therefore it must be objectively named, through scales or numerical systems to ensure a more universal language and interpretation. When colors are ordered, they can be expressed in terms of hue, lightness and saturation(14).

The CIE Lab * system, developed by the L’Éclairage International Commission (CIE), is based on color perception based on three different color receivers (red, green and blue) (3,4).

The L * a * b * color space was created after the opposite color theory, where two colors cannot be green and red at the same time, or yellow and blue at the same time.

L * indicates brightness and a * and b * are the color coordinates. Spectrophotometers and colorimeters measure the reflected light of objects at each wavelength or within specific ranges. It then quantifies the spectral data to determine the object’s color coordinates in the L * a * b * color space and presents the information in numerical terms (4).

Although CIELab is a standard for measuring color changes in teeth whitening research, in this study, it was chose to complement this analysis by using numerical values of the Vita Scale, to facilitate understanding the results obtained from a clinical approach.

This study analyzed the influence on bovine teeth color of soaking in dye solutions during in-office bleaching process using hydrogen peroxide 35%. The teeth were exposed to dye solutions once a day for five minutes. This time is based on time required for swallowing during consumption, which varies around 1.6 seconds (3,7) per swallowing as well as the average volume of liquid consumed daily.

Most related studies used bovine teeth due to similarities to human teeth in morphological aspects and physical-chemical behavior. In addition to ease of retrieval, storage and standardization (4).

Two of the population’s most consumed color drinks were selected. Coffee, which is present on a large number of Brazilians’s eating habits (15,16) and red wine, which had its consumption increased considerably in Brazil (16).

This relationship between coloring drinks and whitening treatment has been extensively studied, but focus lies on the hypothesis that tooth whitening causes changes on enamel’s surface, such as increased surface porosity, demineralization, organic matrix degradation and loss of calcium and phosphate, causing surface microhardness reduction, which would increase susceptibility to staining (10,11).

During a review of current literature, it appears that several laboratorial studies have contributed to clarify the pigmenting effect of dye-rich foods on whitened dental surface, however results are still controversial and conflicting (3,4,7,11-13,17). This increases the need for controlled clinical trials that create safer evidence on this subject.

When evaluating values on CIE Lab * scale, this study revealed that in ΔE comparisons there was no significant influence between the groups tested, in agreement with previous studies findings (3,14). However, disagreeing with the findings of Araújo *et al.* (11), who evaluated color change and tooth enamel mineral loss, as well as influence of pigment solutions commonly used by adolescents undergoing supervised bleaching with 10% carbamide peroxide for 6 hours daily. Concluding that acidic beverages cause enamel mineral loss which can modify tooth surface and interfere with whitening results when consumed concomitantly.

Such divergence can be explained by the difference between bleaching gels used in each research, as well as the storage solution used for the samples (18), because gel’s pH also has an important influence on properties of tooth enamel subjected to whitening, low pH gels may induce in vitro morphological changes in enamel and the presence of human saliva can eliminate demineralization effect caused by low pH of the whitening gels (19).

Regarding whitening speed (Graph 1), it can be noted that there was influence by contact with dyes, groups that had contact with wine 24 hours and 72 hours after whitening session, as well as with coffee 24 hours after whitening session obtained less expressive whitening results between the evaluated times. Another finding that reveals an influence of diet dyes on whitening results speed, can be found on ΔE2, ΔE4 and ΔE8 analyzes (Table 2), where great color variation is revealed when considering immediate post-session and immediate pre-session measurements, where all specimens maintained daily contact with dyes.

Results revealed that frequent contact with red wine, in this study, had a greater influence on reducing whitening effect speed, in agreement with previous studies findings (17), as shows figure 2. However, these changes did not significantly affect the final outcome of treatment. This can be explained by the remineralizing role of artificial saliva between whitening sessions (11).

Regarding whitening results reversal, it is observed on Table 2 and figure 2 that after one week it was more significant on groups that suffered pigmentation by wine (GW24 e GW72), wich presented lower ΔE values one week after bleaching when compared to control group.

A secondary but also important finding for clinical dentistry procedures, lies on comparison between dental colors at times T6 (1 week after whitening before prophylaxis) and T7 (1 week after whitening and after prophylaxis), where variation greater than 1 was observed on all groups. Therefore, an effective dental color registration should always be performed after prophylaxis.

## Conclusions

Contact with dyes during in-office bleaching treatment with 35% hydrogen peroxide did not influence the staining averages after three bleaching sessions. However, whitening effect speed was influenced by contact with coffee from 24 hours after bleaching sessions and with the wine from 24 and 72 hours after whitening sessions.

There was reversion of whitening results after one week for all groups, mainly for groups that came in contact with red wine either 24 or 72 hours after whitening session and coffee after 24 hours.
